# Immobilization of an Invertase Produced by a Bacterium Isolated from the Peach Palm Fruit (*Bactris gasipaes*): Influence of the Support Material and Biochemical Characterization

**DOI:** 10.1021/acs.jafc.6c02898

**Published:** 2026-05-18

**Authors:** Larissa Santos Saraiva, Natanael do Carmo Sousa Gomes, Joicy Silva e Silva, Rafaela Rocha Pinto, Gabriela Eustáquio Lacerda, Maria Olivia dos Santos Oliveira, Alex Fernando de Almeida, Rafael Firmani Perna, Michelle da Cunha Abreu Xavier, Sergio Andres Villalba Morales

**Affiliations:** † Postgraduate Program in Food Science and Technology, Federal University of Tocantins, Palmas, TO 77001-090, Brazil; ‡ Food Engineering Department, Federal University of Tocantins, Palmas, TO 77001-090, Brazil; § Postgraduate Program in Chemical Engineering, Institute of Science and Technology, Federal University of Alfenas, Poços de Caldas, MG 37715-400, Brazil

**Keywords:** invertase, immobilization, fructooligosaccharides, enzyme, Amazonian fruit

## Abstract

This study evaluated the immobilization of an invertase from *Bacillus tequilensis*, isolated from the fruit of the peach palm, on silica gel (SG) and polyhydroxybutyrate (PHB), aiming at its application in sucrose hydrolysis for invert sugar production. Immobilization was performed at 30 °C and pH 5.5 using glutaraldehyde-functionalized supports. Immobilization yield (IY), recovered activity (RA), kinetic parameters, and pH, thermal, storage, and operational stabilities were evaluated. IY values reached 72% (SG) and 77% (PHB), with RA of 35% and 67%, respectively. Both immobilized derivatives exhibited improved storage stability compared to the soluble enzyme. The SG-immobilized invertase demonstrated enhanced thermostability and higher fructose production and retained over 50% of its activity after three reuse cycles. In contrast, the PHB derivative showed greater substrate affinity. These results demonstrate that the *B. tequilensis* invertase immobilized on SG is a promising and stable biocatalyst derived from the Amazonian biodiversity, suitable for sustainable invert sugar production.

## Graphical abstract

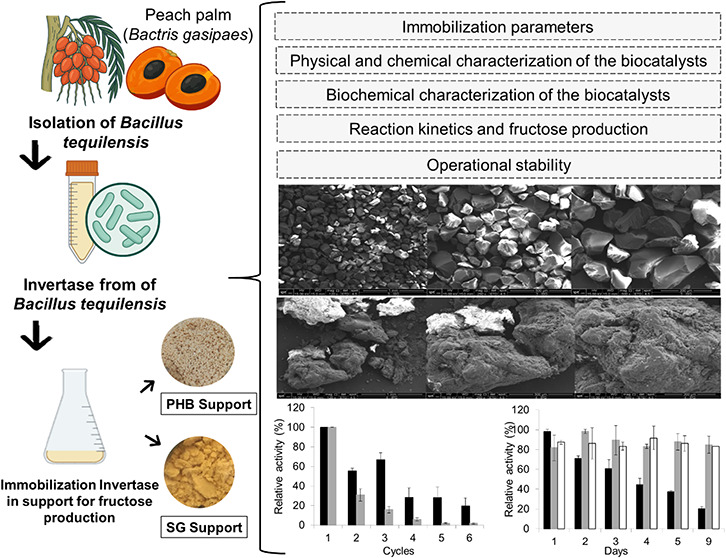

## Introduction

Invertase, or β-fructofuranosidase (FFase, EC 3.2.1.26), is an enzyme that catalyzes sucrose hydrolysis for the production of glucose and fructose, thus forming invert sugar, which is largely important for the industry, since fructose has a higher degree of sweetness than sucrose and does not crystallize during processing^1,2^ Among the numerous applications of this sugar, the production of candies, jellies, molasses for alcoholic beverages, honey, chocolates, liquid coatings, cookies, syrups, nutraceuticals, and animal feed formulations can be highlighted.^3^

Currently, the invertase produced by *Saccharomyces cerevisiae* is the most used in the food industry, given its high catalytic activity and lack of toxicity and pathogenicity.^3,4^ Nevertheless, research works have demonstrated that there is a wide variety of useful microbial sources for invertase production with adequate properties that allow the generation of products that are safe for consumption.^5–7^ In this context of searching for alternative and regionally adapted enzymatic sources, the Amazon biome stands out for providing numerous fruit species that are rich in yeasts and bacteria capable of producing invertase and other enzymes; however, these microbial resources remain underexplored.

In this scenario, invertase-producing strains of *Bacillus tequilensis* isolated from the fruit of the peach palm (*Bactris gasipaes*) constitute a novel and regionally relevant enzymatic source. Unlike conventional yeast invertases, bacterial enzymes may exhibit distinct structural adaptability, environmental tolerance, and catalytic behavior, offering potential advantages under specific bioprocess conditions. However, despite this potential, there are no reports in the literature regarding the immobilization and kinetic characterization of invertase produced by *Bacillus* tequilensis isolated from peach palms, which highlights a scientific gap in the exploration of bacterial enzymes derived from Amazonian biodiversity.

Even when promising enzymatic sources are identified, soluble microbial invertases present a rapid deactivation rate and are not reusable, which limits their application and increases the cost of invert sugar production.^6,8^ Therefore, strategies aimed at enhancing enzyme stability are essential for technological feasibility. The immobilization of this enzyme may increase its thermal and operational stability, allowing its reuse and application under diverse operational conditions.^8,9^ Furthermore, the use of immobilized enzymes facilitates their separation from the reaction medium and allows the use of heterogeneous catalytic reactors.^10^ However, the performance of an immobilized enzyme depends on the immobilization method and the support material employed.^11,12^

Several physical and chemical methods have been reported for invertase immobilization.^13^ Usually, physical methods are more indicated to maintain the catalytic activity of the enzymes, whereas chemical methods are more indicated to maintain stability for long periods of time.^14^ Specifically, adsorption is a physical method that requires hydrophobic or ionic interaction between support and enzyme, being a reversible and simple method that allows the reuse of the support after the inactivation of the immobilized enzyme, resulting in lower costs.^11^ Conversely, covalent binding is an irreversible and highly stable immobilization method that can induce the formation of strong interactions between enzyme and support, thus reducing enzyme desorption and increasing its thermal stability and half-life.^11,15,16^ For this, the support is first treated with a cross-linking agent, such as glutaraldehyde, so that the enzyme and support react with the molecules of this agent, and immobilization occurs by the reaction of amine groups with aldehyde groups of the functionalized support.^17^

Considering that the physicochemical properties of the support directly influence the enzyme orientation, rigidity, and catalytic behavior, the selection of an appropriate matrix becomes a critical factor.^18^ Among the support materials reported for invertase immobilization, polyacrylonitrile/polyaniline composite polymers, chitosan, iron oxide nanoparticles, and beidellite nanoclays stand out.^13,19–22^ Recently, silica gel (SG) and polyhydroxybutyrate (PHB) have stood out for their applicability in the immobilization of fructosyltransferase enzymes, used for the conversion of sucrose into fructooligosaccharides.^23,24^ Silica is a nontoxic hydrophilic material of low cost that presents high thermal stability.^25^ PHB is a hydrophobic material, biodegradable, and renewable, produced by bacteria.^26^

However, comparative studies evaluating how supports with distinct physicochemical characteristics influence the immobilization behavior, kinetic parameters, and stability of bacterial invertases have remained limited. Therefore, this work aimed at evaluating the influence of the support material on the immobilization of the invertase from *Bacillus tequilensis*, isolated from the Amazonian fruit peach palm, as well as the biochemical properties of the immobilized enzyme, with the aim of their application in sucrose hydrolysis.

## Material and Methods

### Materials

The invertase was produced by the bacterial strain *Bacillus tequilensis* (PP6) isolated from the Amazonian fruit of the peach palm. Silica gel (pore and particle sizes of 60 Å and 63–200 μm, respectively) was purchased from Sigma-Aldrich (St. Louis, MO), and polyhydroxybutyrate (PHB) (of bacterial origin and used as an organic support for enzyme immobilization) was provided by the Institute for Technological Research (IPT/SP, São Paulo, Brazil). Yeast extract, sucrose, monopotassium phosphate (KH_2_PO_4_), disodium phosphate (Na_2_HPO_4_), ammonium sulfate ((NH_4_)_2_SO_4_), magnesium sulfate (MgSO_4_), meat peptone, meat extract, and agar were obtained from Labsynth (Diadema, Brazil). Glutaraldehyde solution (25% v/v in water) and ethanol (99% v/v) were purchased from Dinâmica (Diadema, Brazil). All chemicals used in this study were of analytical grade.

### Pretreatment and Functionalization of the Supports

An amount of 50 mL of ethanol (99% v/v) was added to 10 g of polyhydroxybutyrate (PHB). The system was agitated at 50 rpm and 25 °C for 2 h. The support was then separated by filtration, washed with distilled water, and dried for 24 h in an oven at 80 °C.^23^ In turn, silica gel (SG) underwent the step of drying in an oven at 80 °C for 24 h.

For support functionalization, 18 mL of 25% glutaraldehyde (v/v) (Dinâmica Química Contemporânea Ldta, Brazil) was added to 2 g of support. The support systems and the functionalizing agent were agitated for 18 h at 50 rpm and 25 °C, subsequently being vacuum-filtered and dried in a desiccator for 24 h.^23^

### Invertase Production

The invertase was produced from the bacterial strain *Bacillus tequilensis* (PP6), isolated from Amazonian peach palm fruits. The bacterium was preserved in cryotubes containing a cryoprotective solution composed of 50% (v/v) sterile glycerol solution (40% w/v) and 50% (v/v) nutrient broth (5 g/L meat peptone and 3 g/L meat extract) and stored at −80 °C until reactivation. For plate subculturing, nutrient agar medium was prepared containing 5 g/L meat peptone, 3 g/L meat extract, and 20 g/L agar. The medium and Petri dishes were sterilized by autoclaving at 121 °C for 15 min. After sterilization, streaking was performed using sterile disposable inoculation loops. The inoculated plates were incubated in a biochemical oxygen demand (BOD) incubator at 30 °C for 24 h. After microbial growth on plates, a preinoculum was prepared in 125 mL Erlenmeyer flasks containing 20 mL of basal medium composed of 5 g/L peptone and 3 g/L meat extract. The medium was sterilized at 121 °C for 15 min and inoculated with a single colony using sterile disposable loops. The preinoculum was maintained under agitation (150 rpm) for 24 h at 30 °C. For submerged cultivation, 2 mL of preinoculum was transferred into 18 mL of sterile mineral medium containing 1.5 g/L KH_2_PO_4_, 3.5 g/L Na_2_HPO_4_, 2 g/L (NH_4_)_2_SO_4_, 1 g/L yeast extract, 0.2 g/L MgSO_4_, and 20 g/L sucrose in each Erlenmeyer flask. The culture was maintained at 150 rpm for 48 h at 30 °C. After incubation, samples were transferred to 15 mL Falcon tubes and centrifuged at 3000 rpm for 30 min at 10 °C, obtaining the crude extracellular enzymatic extract (supernatant) for subsequent immobilization.

### Invertase Immobilization

In Erlenmeyer flasks (125 mL), 1 g of support was added to 20 mL of the crude enzyme extract. The samples were placed in a Dubnoff bath and agitated at 175 rpm and 30 °C for 8 h, and aliquots of 1 mL of the liquid phase (supernatant) were collected every hour, immediately centrifuged to remove suspended particles when necessary, and stored at 4 °C until enzymatic activity determination. After this period, the support was vacuum-filtered, thus producing the immobilized derivative. The samples were stored under refrigeration for further analysis of enzymatic activity and stability assays.

### Determination of the Immobilization Parameters

The parameters of immobilization yield (IY) and recovered activity (RA) were determined according to Tavernini et al.,^27^ using eqs 1 and 2.

1 $$\mathrm{IY}\;\left(\%\right)=\frac{A_{\mathrm{ti}}-A_{\mathrm{tf}}}{A_{\mathrm{ti}}}\times 100$$

2 $$\mathrm{RA}\;\left(\%\right)=\frac{A_{\mathrm{td}}}{A_{\mathrm{ti}}-A_{\mathrm{tf}}}\times 100$$

where *A*_td_ is the enzymatic activity (U) of the immobilized biocatalyst, *A*_ti_ is the initial enzymatic activity of the free enzyme before immobilization, and *A*_tf_ is the enzymatic activity remaining in the supernatant after immobilization. Therefore, *A*_ti_ – *A*_tf_ represents the amount of enzyme effectively immobilized, and RA expresses the fraction of enzymatic activity retained in the immobilized biocatalyst relative to the activity effectively immobilized.

### Determination of the Enzymatic Activity

The determination of the enzymatic activity (U/mL for soluble enzyme and U/g for immobilized enzyme) was performed by employing the method of Miller,^28^ where 3,5-dinitrosalicylic acid (DNS) is reduced by the reducing sugar, in an alkaline medium, to 3-amino-5-nitrosalicylic acid. During this reaction, the aldehyde group is oxidated to carboxylic acid, resulting in a change in sample coloration from yellow to brown-red. This analysis was performed in duplicate, starting from the preparation of the reaction medium using the buffer McIlvaine pH 5.0 and 2% (w/v) of sucrose. 800 μL of the reaction medium was placed in a water bath at 50 °C for 5 min, and after this time, 200 μL of the soluble enzyme sample or 0.2 g of the immobilized biocatalyst was added to this reaction medium. After homogenization, 200 μL of aliquots was collected from the liquid reaction mixture and added to 200 μL of DNS, which was regarded as time zero. After 5 min, another aliquot of 200 μL of the liquid reaction mixture with the same sample was added to the DNS solution. These samples were taken to the water bath at 100 °C for 5 min, and immediately after, they were placed in an ice bath, with the addition of 2 mL of distilled water. The absorbance readings were performed in a UV–vis spectrophotometer at 540 nm. The values were used to calculate the enzymatic activity (U/mL or U/g) and the mean of the duplicates. One unit of enzyme (U) is the quantity of enzyme necessary for the production of 1 μmol per minute of reducing sugar at 50 °C and pH 5.0, per gram (g) of immobilized enzyme or milliliter (mL) of soluble enzyme.^29^

### Physical and Chemical Characterization of the Functionalized Supports and Immobilized Biocatalysts

The morphological properties of the supports after glutaraldehyde functionalization and prior to enzyme immobilization were analyzed by scanning electron microscopy (SEM) using an electron microscope (Zeiss EVO MA-10, Germany), at an accelerating voltage of 15 kV and distances of 15 mm. The specific surface areas were determined by nitrogen physisorption at 77 K in a Micromeritics Gemini VII analyzer, using the BET method,^30^ and the volume and size of the pores were obtained using the BJH method.^31^ Furthermore, the characterization of the chemical bonds present in the immobilized biocatalyst was determined by infrared spectroscopy (FT-IR) using a spectrometer (Agilent Cary 630, United States) operating at a range from 650 to 4000 cm^–1^.

### Biochemical Characterization of the Immobilized Invertase

#### pH Stability Assays

For the pH stability assays, the enzyme immobilized on SG and PHB was incubated in MCIlvaine buffer in the absence of substrate, with the pH adjusted to 4.5, 5.0, 5.5, 6.0, and 6.5 at 4 °C for 24 h. After this period, the enzymatic activity of the biocatalyst was determined.

#### Storage Stability

The storage stability assays were performed for 9 days, conducted with the enzyme immobilized on SG and PHB, using the soluble enzyme as a control. The samples were stored under refrigeration at 4 °C, and the enzymatic activity was determined daily for the first 5 days and then sporadically, at regular intervals, until day 9.

#### Thermal Stability Assays and Thermodynamic Parameters

The enzyme immobilized on functionalized SG and PHB was incubated in MCIlvaine buffer (156 Mm), pH 5.0, in the absence of the substrate, at different temperatures (30, 40, 50, and 60 °C). The samples were collected at different time intervals (60, 120, 240, 360, and 1440 min) for the determination of the enzymatic activity.^23^ The first-order thermal deactivation constant (*K*_d_, in min^–1^) was estimated (eq 3).

3 $$\ln A={\ln A}_{0}-K_{d}t$$

in which *A* (U g^–1^) refers to the enzymatic activity, *A*_0_ (U g^1^) to the initial enzymatic activity, and *t* to the incubation time (min).

The half-life time (*t*_1/2_ in min) of the biocatalyst was calculated using eq 4.^24,32^

4 $$t_{1/2}=\frac{\ln2}{K_{d}}$$

To calculate the activation energy for thermal denaturation (*E*_d_, in kJ mol^–1^), the linearization of the Arrhenius equation was used (eq 5).^33^

5 $$\ln\left(K_{d}\right)=-\frac{E_{d}}{R}\frac{1}{T}+\ln\left(A\right)$$

in which *R* is the universal gas constant (8.314 J/mol K), *T* is the absolute temperature (K), and *A* is the Arrhenius frequency factor.

The variations in enthalpy (Δ*H*_d_ in kJ mol^–1^), Gibbs energy (Δ*G*_d_, in kJ mol^–1^), and entropy (Δ*S*_d_ in kJ mol^–1^ K^–1^) of activation for the thermal denaturation were calculated using eqs 6, 7, and 8, respectively.^24,34^

6 $$\Delta H_{d}=E_{d}-\mathrm{RT}$$

7 $$\Delta G_{d}=-\mathrm{RT}\cdot \ln\left(\frac{K_{d}h}{K_{B}T}\right)$$

8 $$\Delta S_{d}=\frac{\Delta H_{d}-\Delta G_{d}}{T}$$

in which *h* is Planck’s constant (11.04 × 10^–36^ J/min) and *K*_B_ is the Boltzmann constant (1.38 × 10^–23^ J/K).

#### Determination of the Reaction Kinetics and Fructose Production

For the kinetics analysis, 1.0 g of the enzyme immobilized on SG and PHB was added to 4.0 mL of the reaction medium containing MCIlvaine buffer (156 mM and pH 5.0) at 50 °C and different sucrose concentrations (5 g/L, 10 g/L, 20 g/L, 50 g/L, and 100 g/L), for 1 h. Aliquots of the reaction medium were taken for the analysis of the concentration of reducing sugars. The Michaelis–Menten model (eq 9) was adjusted to the data obtained by nonlinear regression, to determine the kinetic parameters: maximum velocity of the reaction (*V*_max_) and Michaelis–Menten constant (*K*_m_).^24^

9 $$V=\frac{V_{\max}\left[S\right]}{\left[S\right]+K_{m}}$$

where *V* is the initial reaction velocity and [*S*] is the substrate concentration.

The production of fructose catalyzed by the invertase immobilized on SG and PHB was evaluated in 4.0 mL of the reaction medium containing MCIlvaine buffer (156 mM and pH 5.0) at 50 °C for 8 h, using 100 g/L of sucrose. Fructose concentration as a function of time was obtained by DNS analysis.

#### Operational Stability Assays

The operational stability of the enzyme immobilized on SG and PHB was evaluated during six sequential reaction cycles, where 1 g of the biocatalyst was added to 4.0 mL of the reaction medium (McIlvaine buffer, pH 5.0, and 2% (w/v) sucrose) at 50 °C. After each 1 h cycle of the reaction, the biocatalyst was separated from the reaction medium by filtration and used in a new cycle under the same conditions. 200 μL of the filtered medium was added to 200 μL of DNS and taken to the water bath at 100 °C for 5 min. After this period, the sample was subjected to an ice bath, with the addition of 2.0 mL of distilled water. Absorbance reading was performed in a spectrophotometer at 540 nm, and the results were used to determine the concentrations of the reducing sugars.^24^

### Statistical Analysis of the Data

The results obtained were analyzed using the test of means and subjected to the analysis of variance (ANOVA) and to Tukey’s test (5% of significance), using the software Sisvar. The graphs were performed using the program Origin Pro 2017, and the analyses were conducted in triplicate.

## Results and Discussion

### Physicochemical Characterization of Biocatalysts

The morphology of the SG and PHB particles is observed in the micrographs shown in Figure 1A,B, respectively. The SEM analysis was performed as a qualitative assessment of the surface morphology. It can be observed that PHB particles present a more irregular and rough surface compared with SG particles, which may contribute to increased enzyme–support interaction through a larger external contact area.

**Figure 1:**
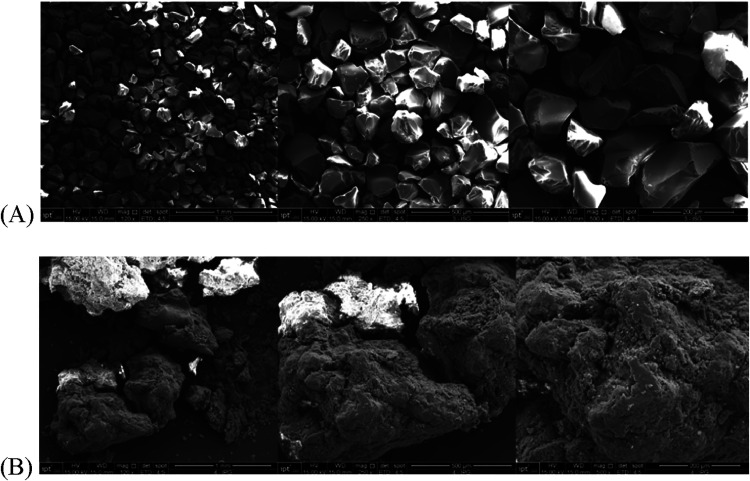
Electron micrographs of the supports, SG (A) and PHB (B).

The textural parameters obtained by nitrogen physisorption (BET and BJH methods) are presented in Table 1. Silica gel exhibited a significantly higher specific surface area compared to PHB, whereas both supports presented pore diameters within the mesoporous range (2–50 nm) according to IUPAC classification.^35^ Mesoporosity is particularly relevant for enzyme immobilization, since pore sizes comparable to or larger than the hydrodynamic diameter of the enzyme may favor enzyme accommodation while minimizing steric hindrance.

**Table 1:** Characterization of the Functionalization Particles

support	specific surface area (m^2^/g)	pore size (Å)	pore volume (cm^3^/g)
SG	211.09	21.33	7.92 × 10^–2^
PHB	4.36	20.36	1.33 × 10^–3^

The higher surface area of SG suggests a greater number of available anchoring sites for covalent attachment after glutaraldehyde functionalization, which may contribute to stronger multipoint interactions and an enhanced structural stabilization of the enzyme. In addition, mesoporous structures may influence substrate diffusion and internal mass transfer phenomena.^35^ Supports with larger pore volumes and adequate pore diameters tend to reduce diffusional resistance, facilitating substrate accessibility to the active site and product release.

In contrast, PHB presented a lower specific surface area and distinct pore characteristics, which may limit internal diffusion and favor immobilization predominantly on the external surface of the particles. Such differences in textural properties may partially explain the observed variations in the recovered activity, fructose production, and operational stability between SG and PHB derivatives. Diffusional limitations and differences in enzyme orientation on hydrophilic (SG) versus hydrophobic (PHB) matrices may influence the effective catalytic turnover under the prolonged hydrolysis conditions.

The FT-IR spectra of the immobilized derivatives (Figure 2) exhibit broad absorption bands around 3300 cm^–1^, which may be attributed to N–H stretching vibrations of protein amine groups overlapping with O–H stretching from hydroxyl groups present on the support surfaces and adsorbed moisture.^36^ Such broadening is commonly observed in enzyme-immobilized systems and reflects the superposition of multiple hydrogen-bonded functional groups.

**Figure 2:**
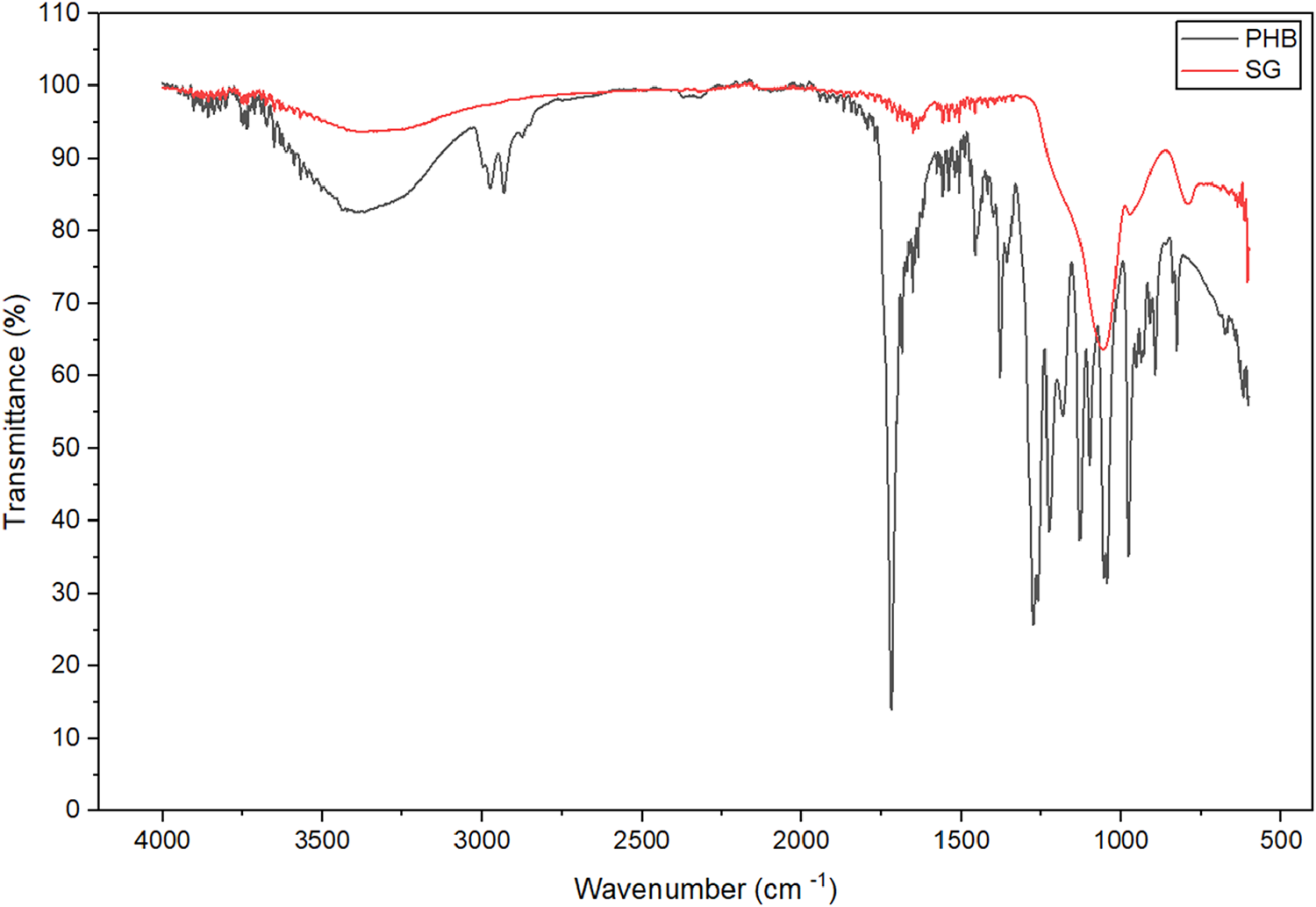
FT-IR of the supports SG and PHB functionalized after the immobilization of the bacterial invertase.

In the region between 1630 and 1650 cm^–1^, bands associated with amide I (C=O stretching of peptide bonds) were detected. In glutaraldehyde-activated systems, this spectral region may also contain contributions from C=N stretching vibrations resulting from Schiff-base formation between aldehyde groups of glutaraldehyde and primary amine groups (e.g., lysine residues) of the enzyme. According to the literature, covalent immobilization mediated by glutaraldehyde involves nucleophilic attack of protein amines on aldehyde groups, forming imine linkages (C=N), whose absorption may overlap with amide I bands, complicating definitive assignment.^16,37^ Therefore, although the spectral changes observed are consistent with chemical interaction between enzyme and support, FT-IR alone does not provide unequivocal proof of exclusive covalent bonding.

For the PHB-based derivative, characteristic ester C=O stretching vibrations around 1720 cm^–1^ and C–O–C bands between 1050 and 1250 cm^–1^ were preserved after immobilization, indicating maintenance of the polymer backbone. Due to the hydrophobic and semicrystalline nature of PHB,^38,39^ enzyme immobilization may involve both covalent bonding, promoted by glutaraldehyde functionalization, and physical adsorption driven by hydrophobic interactions. These interactions may influence enzyme orientation, as hydrophobic surfaces can induce preferential conformations that either expose or partially shield the active site. Furthermore, such interactions may contribute to diffusional limitations and microenvironmental effects, which can affect the catalytic behavior of immobilized enzymes.

In contrast, silica gel presents a predominantly hydrophilic and polar surface rich in silanol groups (Si–OH), which may facilitate different interaction patterns, including hydrogen bonding and enhanced substrate accessibility. The distinct surface chemistries of SG and PHB likely contribute to the differences observed in catalytic behavior and operational stability.^40^

### Invertase Immobilization Parameters

The enzymatic activity of invertase in the crude enzyme extract, in the presence and absence of the supports, is presented in Figure 3. It was observed that the activity of the soluble enzyme decreased more rapidly in the presence of both supports than in its absence (control sample). Possibly, the soluble enzyme migrates from the reaction medium to the surface of the supports, indicating enzyme immobilization. Nonetheless, the loss of enzymatic activity during immobilization does not always indicate that the enzyme is being immobilized, since soluble enzymes may present a rapid thermal deactivation under the immobilization conditions.^41^ In the case of the invertase from *B. tequilensis*, a significant drop in its activity is also observed in the absence of the supports at 8 h of immobilization, indicating that higher immobilization times would be detrimental to preserving enzymatic activity.

**Figure 3:**
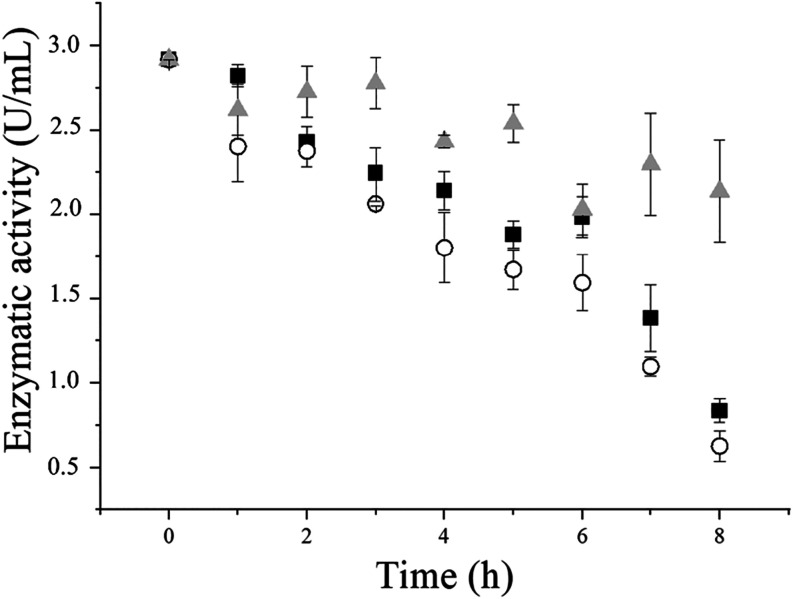
Kinetic profile of the immobilization of the invertase from *B. tequilensis* (PP6) for 8 h at 30 °C on SG (■) and PHB (○) and activity of the soluble enzyme without the presence of any support (gray triangle).

Araújo et al.^23^ immobilized a fructosyltransferase in both functionalized and nonfunctionalized PHB for the period of 8 h, obtaining enzymatic activities of 50% and 60% of the initial value observed for the enzyme extract, respectively.

Mishra et al.^42^ immobilized the invertase produced by the yeast *Saccharomyces cerevisiae*, using silica-based supports for 12 h, which was enough time to achieve the maximum enzyme binding. It is emphasized that studies on the immobilization of bacterial invertase on SG and PHB have not been reported.

After 8 h of invertase immobilization, values of 72.24 ± 2.36% and 76.72 ± 3.10% were obtained for the IY of SG and PHB, respectively, and values of 34.62 ± 3.61% and 60.67 ± 4.07% were obtained for the RA of SG and PHB, respectively. These results indicate that both supports present a great potential for the immobilization of the invertase from *B. tequilensis* (PP6). IY is related to the percentage of enzyme that was immobilized in relation to the enzyme present in the medium initially, and RA indicates the percentage of activity that the enzyme presents after its immobilization in the support.^41^ Nonetheless, the invertase immobilized on PHB presented a significantly higher RA, suggesting greater preservation of catalytic conformation during the immobilization process. Considering that the supports were previously functionalized with glutaraldehyde, the results are consistent with enzyme–support interactions promoted by this cross-linking agent although exclusive covalent bonding was not experimentally confirmed.^17,43^

Rasbold et al.^44^ found 98% of immobilization yield for the invertase produced by the fungus *Cunninghamella echinulata* and immobilized on calcium alginate. The high immobilization yield obtained by the authors can be explained by the fact that the enzyme had been previously purified. Araújo et al.^23^ used PHB functionalized with glutaraldehyde to immobilize a fructosyltransferase by the method of adsorption and covalent bonding and obtained an immobilization yield of approximately 55% and recovered activity of 11% under the same conditions as in this study, explaining that immobilization yield may be affected by impurities in the crude extract, including amino acids, low-weight polypeptides, and carbohydrates, substances that may possibly interact with the enzyme and the support.

It is important to emphasize that the enzyme preparation used in this study consisted of a crude extracellular extract and was not subjected to purification prior to being immobilized. Therefore, the immobilization efficiency was evaluated based on enzymatic activity recovery (IY and RA), which reflects the catalytically active fraction effectively retained on the support. Crude extracts may contain additional proteins and low-molecular-weight compounds capable of interacting with the support surface, potentially influencing the immobilization yield. Nevertheless, the retained catalytic performance observed for both supports suggests that the active invertase fraction was successfully immobilized.

### pH Stability

The pH stability results regarding the incubation of the invertase immobilized on PHB and SG functionalized with glutaraldehyde are presented in Figure 4. The enzyme immobilized on PHB demonstrated greater stability at pH values between 5.0 and 6.0, whereas at pH 4.5 and pH 6.5, it retained approximately 50% of its activity. On the other hand, the enzyme immobilized on SG presented its highest stability at pH 5.0 and an activity reduction of approximately 75% at pH 5.5 and pH 6.5, indicating a smaller pH stability range.

**Figure 4:**
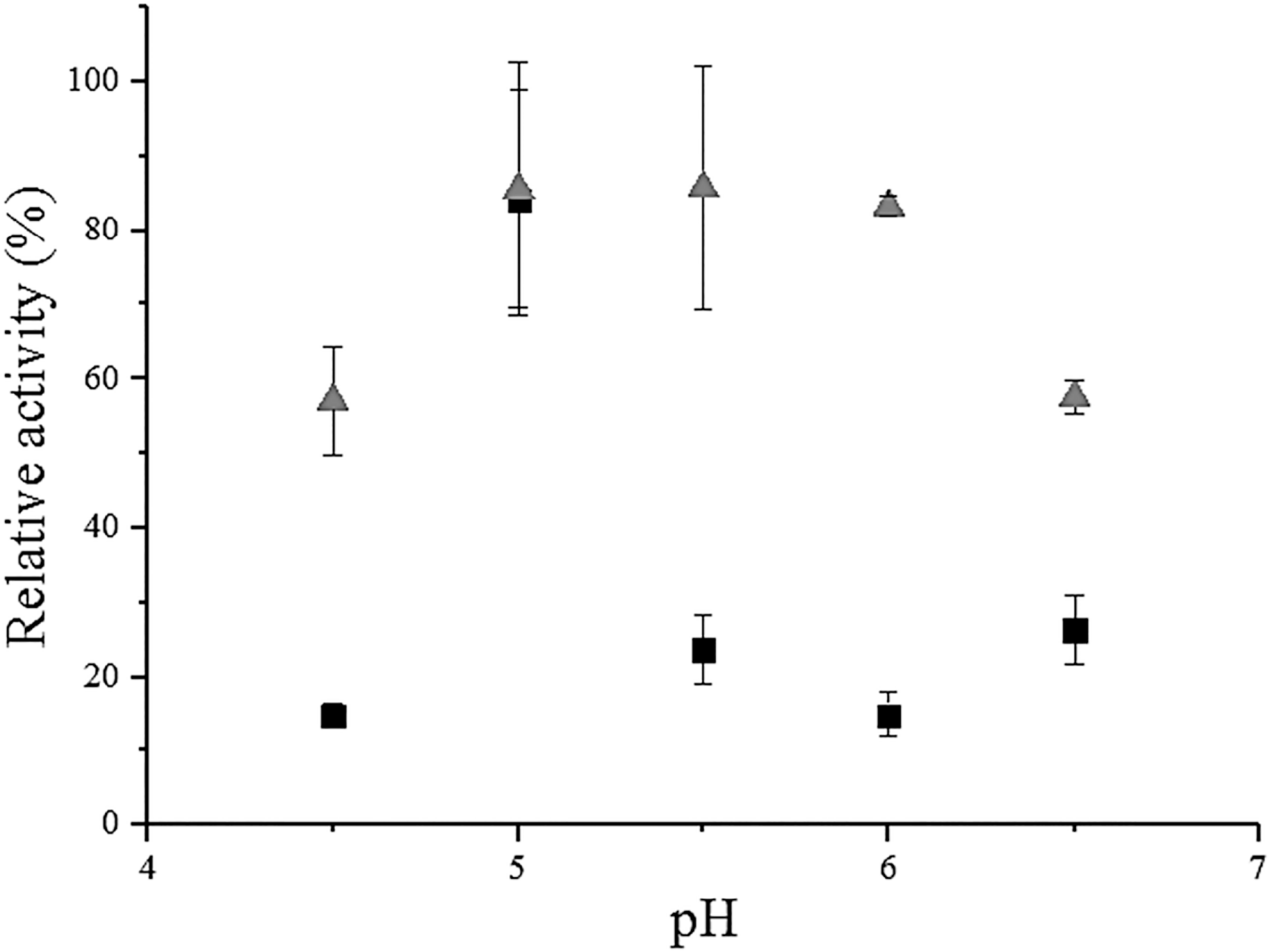
Stability of the invertase from *B. tequilensis* (PP6) immobilized on functionalized SG (■) and PHB (gray triangle) after 24 h of incubation at 4 °C at different pH values, with the maximum enzymatic activity of 2.30 U/g.

Immobilization tends to protect the structure of the enzyme from the effects of pH; thus, higher pH stability favors storage and industrial applications.^45^ As observed for thermal stability, the samples immobilized on functionalized PHB presented a similar stability to that of the soluble enzyme; therefore, this support may be offering a favorable environment to the enzyme.^42^ The fact that the stability of the enzyme immobilized on the functionalized silica gel is remarkably higher at pH 5.0 may be related to substrate diffusion limitation, since, as the microenvironment and the tertiary structure of the enzyme are altered in the covalent bond, the activity decreases at certain pH values.^46^ When the support does not have ionic strength and the enzyme molecules get immobilized on the external surface, the pH of the reaction medium tends not to undergo alterations.^47^

Rasbold et al.^44^ verified the increase in stability in alkaline and acidic pH ranges for invertase immobilization in chitosan activated with glutaraldehyde compared with the soluble enzyme since the formation of the covalent bond in different points of the support guarantees the stability of the tridimensional structure of the biomolecule, as in the immobilization with PHB.

In addition to the structural effects promoted by immobilization, microenvironmental effects associated with the surface chemistry of the supports may also influence the differences in pH stability between SG and PHB. The hydrophilic character of silica gel and the presence of surface silanol groups may modify the local ionic environment surrounding the immobilized enzyme, potentially affecting the ionization state of amino acid residues near the catalytic site.^48^ Such local p*K*_a_ shifts have been reported in immobilized enzyme systems and may contribute to the distinct pH stability profiles observed for SG and PHB derivatives.

### Storage Stability

The storage stability of invertase, both soluble and immobilized on the functionalized SG and PHB, is presented in Figure 5.

**Figure 5:**
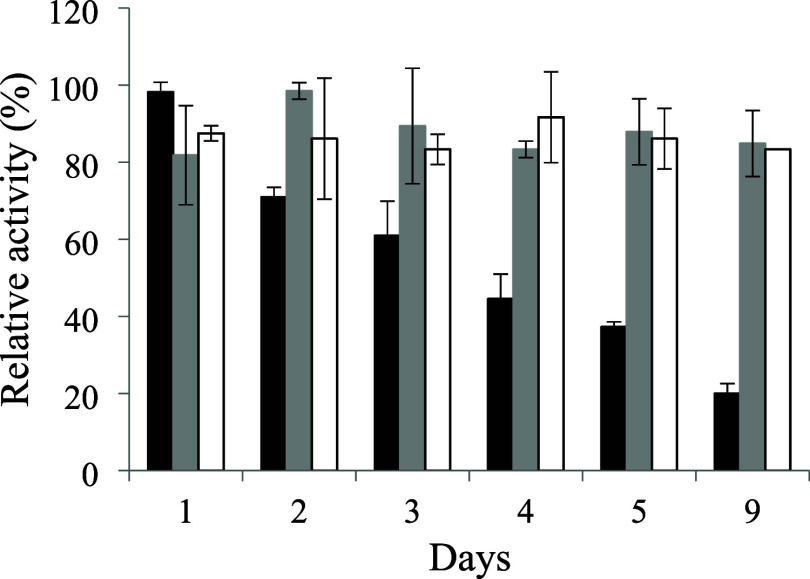
Storage stability of the bacterial invertase, both soluble (■) and immobilized on SG (gray box) and PHB (□), for 9 days at 4 °C.

The enzymatic activity of the soluble invertase demonstrated an expressive drop from the second day of storage, whereas the immobilized samples maintained their activities close to the initial values. After 9 days of storage at 4 °C, the soluble enzyme lost approximately 80% of its initial enzymatic activity, while the immobilized samples continued with values similar to the initial values. This behavior demonstrates that the immobilization of the enzyme in both supports allowed its stability during the 9 days of storage. This result may be attributed to the fact that the functionalization of the supports with glutaraldehyde provides strong and lasting bonds, avoiding the loss of enzymatic activity by process reversibility.^49^

Mishra et al.^42^ evaluated the storage stability of an invertase immobilized on a biohybrid support composed of silica nanoparticles and *Ocimum basilicum* seed, in which the immobilized enzyme presented around 45% and 78% of the activity after 60 days, whereas the free enzyme lost all activity in 25 days. Cabrera et al.^50^ determined the storage stability of the invertase immobilized in magnetic nanoparticles of diatomaceous earth, observing the loss of 72% of activity of the free enzyme; after 120 days, the free enzyme completely lost its activity, while the immobilized invertase retained 83% of activity at the end of this period.

### Thermal Stability and Thermodynamic Parameters

Figure 6 shows the thermal deactivation profiles for the invertase of *B*. *tequilensis* (PP6) immobilized on PHB (Figure 6A) and silica gel (Figure 6B) and incubated at different temperatures. The thermal stability profiles for the invertase immobilized in silica gel demonstrated a decrease in the residual activity as a function of temperature (30, 40, 50, and 60 °C) and incubation time, indicating the thermal denaturation of the biocatalyst. Conversely, it was observed that the enzyme immobilized on PHB, after 24 h of incubation, retained a higher enzymatic activity in relation to the invertase immobilized on silica gel, which achieved values of approximately 51% (1.03 ± 0.05 U g^–1^), 52% (1.05 ± 0.30 U g^–1^), and 45% (0.92 ± 0.14 U g^–1^) at 30, 40, and 50 °C, respectively. At 60 °C, low values of residual activity were obtained for the biocatalyst immobilized on both supports. The exposure of enzymes to high temperatures results in the irreversible loss of their catalytic properties deriving from the cleavage of noncovalent interactions and conformational changes.^51,52^

**Figure 6:**
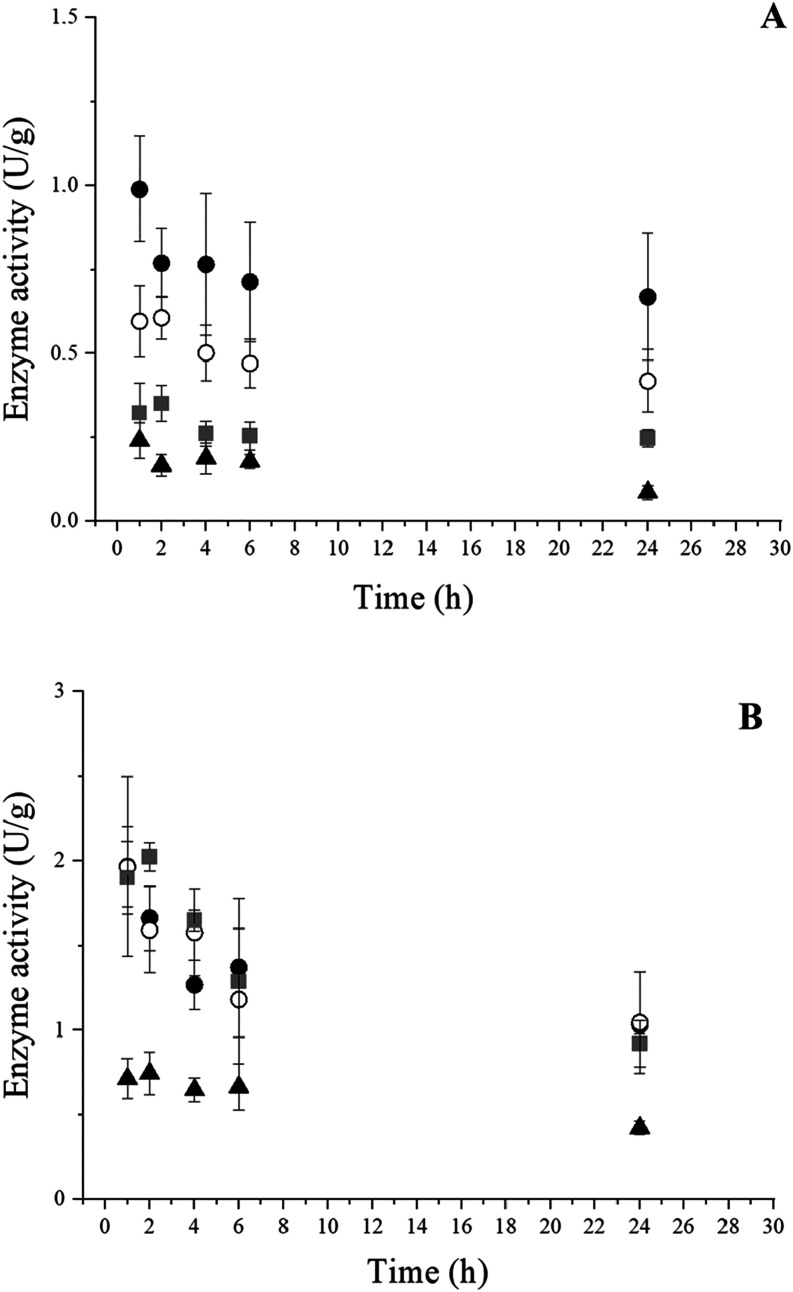
Thermal stability of the invertase from *B. tequilensis* (PP6) immobilized on SG (A) and PHB (B) throughout 24 h of incubation at different temperatures: 30 °C (●), 40 °C (○), 50 °C (gray box), and 60 °C (▲).

Waifalkar et al.^53^ demonstrated a higher thermal stability at temperatures above 60 °C for a commercial invertase immobilized in magnetic nanoparticles coated with chitosan, when analyzing temperatures from 30 to 80 °C. Lorenzoni et al.^54^ immobilized an invertase on chitosan particles, analyzing the thermal stability from 50 to 60 °C, whose results demonstrated that, after 200 h, the biocatalyst retained 83% and 50% of its initial activity at 50 and 60 °C, respectively.

The thermodynamic parameters of the invertase immobilized on the functionalized PHB and silica gel are shown in Table 2. The half-life time (*t*_1/2_) of the enzyme immobilized on both supports decreased, and the first-order thermal deactivation constant (*K*_D_) increased with incubation temperature, indicating irreversible thermal denaturation of the biocatalyst. The *t*_1/2_ is defined as the time required for the enzyme to reduce to 50% of its initial activity at a given temperature.^55,56^ The invertase immobilized on silica gel presented higher *t*_1/2_ values at 30 °C (2.0 times) and 50 °C (1.4 times) compared to the enzyme immobilized on PHB at similar temperatures. Long half-life times indicate that the biocatalyst can stand the effects of the temperature of the medium for a long period, and therefore, it is a parameter that indicates a greater thermostability of the enzyme and its feasibility for industrial application.^24,33,57^

**Table 2:** Thermodynamic Parameters of the Invertase from *B. tequilensis* (PP6) Immobilized on PHB and SG for Different Incubation Temperatures

		temperature (°C)			
parameters	immobilization	30	40	50	60
*K*_d_ (min^–1^)	PHB	4.0 × 10^–4^	4.0 × 10^–4^	7.0 × 10^–4^	5.0 × 10^–4^
	SG	2.0 × 10^–4^	4.0 × 10^–4^	5.0 × 10^–4^	1.0 × 10^–3^
*t*_1/2_ (min)	PHB	1733	1733	990	1386
	SG	3466	1733	1386	693
*E*_d_ (kJ/mol)	PHB	10.48			
	SG	42.39			
Δ*H*_d_ (kJ/mol)	PHB	7.96	7.88	7.80	7.71
	SG	39.87	39.79	39.70	39.62
Δ*G*_d_ (kJ/mol)	PHB	104.31	107.83	109.86	114.27
	SG	106.05	107.83	110.76	112.36
Δ*S*_d_ (kJ/mol K)	PHB	–0.317	–0.319	–0.315	–0.319
	SG	–0.218	–0.217	–0.219	–0.218

Table 2 also shows that the *E*_D_ value of the invertase immobilized on silica gel (42.39 kJ mol^–1^) was approximately four times higher than that observed for the enzyme immobilized on PHB (10.48 kJ mol^–1^), indicating greater resistance to thermal inactivation.^23,58^ Immobilization on glutaraldehyde-functionalized inorganic supports may promote multipoint enzyme–support interactions, in which multiple amino groups of the enzyme react with aldehyde groups on the support surface.^37^ This multipoint attachment can reduce conformational flexibility of the protein, increasing structural rigidity and limiting the structural rearrangements required for thermal unfolding. As a consequence, a greater amount of activation energy is required for denaturation, resulting in higher *E*_D_ values.^23,59^ This effect is consistent with previous reports indicating that intense multipoint covalent attachment may enhance enzyme stability by increasing structural rigidity although it may also lead to reduced catalytic activity due to conformational restrictions.^60^

In addition, the hydrophilic and porous nature of silica gel, characterized by the presence of surface silanol (Si–OH) groups, may contribute to the formation of a stabilizing microenvironment and preservation of the enzyme hydration layer.^25^ Maintenance of this hydration shell has been associated with improved structural stability and resistance to heat-induced unfolding in immobilized biocatalysts. Studies on the thermal stability of the enzyme fructosyltransferase of *Aspergillus oryzae* IPT-301, although this comparison is used here as an analogy, since both enzymes belong to the same functional class of β-fructofuranosidases, demonstrated *E*_D_ values equal to 50.8 kJ mol^–1^ and 56.8 kJ mol^–1^ for the biocatalyst immobilized on polyhydroxybutyrate functionalized with glutaraldehyde and on silica gel, respectively.^23,24^

On the other hand, E_D_ is directly related to the variation in the thermal deactivation enthalpy (Δ*H*_d_), and this is an important thermodynamic parameter associated with the total amount of energy required for biocatalyst denaturation.^57,61^ Table 2 displays high and positive values for Δ*H*_d_ for all incubation temperatures investigated, indicating the thermostability of the invertase immobilized in both supports since a greater amount of energy must be required to stretch, compress, or break the enzyme bonds to change it from its native state to the denatured state.^61,62^ Faria et al.^24^ obtained Δ*H*_d_ values of approximately 54 kJ mol^–1^ for a microbial fructosyltransferase immobilized on silica gel, whereas Araújo et al.^23^ reported values around 48 kJ mol^–1^ for the same enzyme immobilized on polyhydroxybutyrate functionalized with glutaraldehyde.

Although high Δ*H*_d_ values evidence enzyme thermostability, it is also necessary to evaluate the variations in the Gibbs energy (Δ*G*_d_) and the entropy (Δ*S*_d_) of activation for the thermal denaturation. The parameter (Δ*G*_d_) is more precise and reliable for the evaluation of biocatalyst thermostability since it is associated with the enthalpic and entropic contributions.^55,57^ High Δ*G*_d_ values for the invertase indicated that the immobilization on PHB and silica gel gave the enzyme a greater stability, whereas the positive values of this parameter demonstrated that the process of thermal denaturation of the biocatalyst is thermodynamically nonspontaneous. The results obtained in this study corroborate the values of Δ*G*_d_ reported in the literature.^23,24,63^

Finally, the parameter Δ*S*_d_ indicates the amount of energy involved in the transition from the native to the denatured state of the biocatalyst.^55,57^ Table 2 demonstrates negative values of Δ*S*_d_ for the incubation temperatures investigated, indicating the transition of the enzyme to a more ordered state. This behavior may be associated with increased structural rigidity of the immobilized enzyme.

In this context, the resistance of the biocatalyst to unfolding may be associated with stronger hydrophobic interactions, while weakened polar interactions at high temperatures may favor structural destabilization. Furthermore, the low Δ*S*_d_ values indicate that thermal inactivation did not cause a relevant alteration in the tertiary structure of the invertase; in other words, most of the hydrogen bonds responsible for maintaining the active structure of the catalytic sites of the biocatalyst are still present in the activated complex.^64^

### Reaction Kinetics

It is possible to observe that the activity of the immobilized enzyme increased with the rise in substrate concentration, adjusting to a behavior described by the Michaelis–Menten kinetics and indicating that at smaller concentrations, the amount of substrate was insufficient to perform the enzymatic reaction.

The kinetic parameters obtained by the adjustment of the Michaelis–Menten model to the experimental data on enzymatic activity are demonstrated in Figure 7. The reactions catalyzed by the enzyme immobilized on PHB and SG presented *V*_max_ values close to each other, indicating a similar potential of application for both catalysts. Low *V*_max_ values may imply that the functionalizing agent does not provide the effect necessary for a good binding of the enzyme to the support, and high values indicate the need for less substrate to convert into product.^65^ On the other hand, the *K*_m_ value was smaller for the immobilization on PHB, indicating a higher affinity of the enzyme for the substrate, since the ratio of *V*_max_ and *K*_m_ would also be smaller for this immobilized enzyme (Table 3). The immobilization process may increase the *K*_m_ value, since it leads to alterations in the structure of the immobilized enzyme, because of the addition of functionalizing agents and diffusional restrictions, thus affecting the access of the substrate to the active sites of the enzyme, besides the irregular surface presented by the supports.^55,66^

**Table 3:** Kinetic Parameters Obtained by the Adjustment of the Michaelis–Menten Model for Sucrose Hydrolysis Catalyzed by the Bacterial Invertase Immobilized on the Functionalized PHB and SG

support	*V*_max_ (U/g)	*K*_m_ (g/L)	*R* ^2^
PHB	0.40 ± 0.02	2.40 ± 0.92	0.77
SG	0.42 ± 0.07	11.19 ± 4.38	0.85

**Figure 7:**
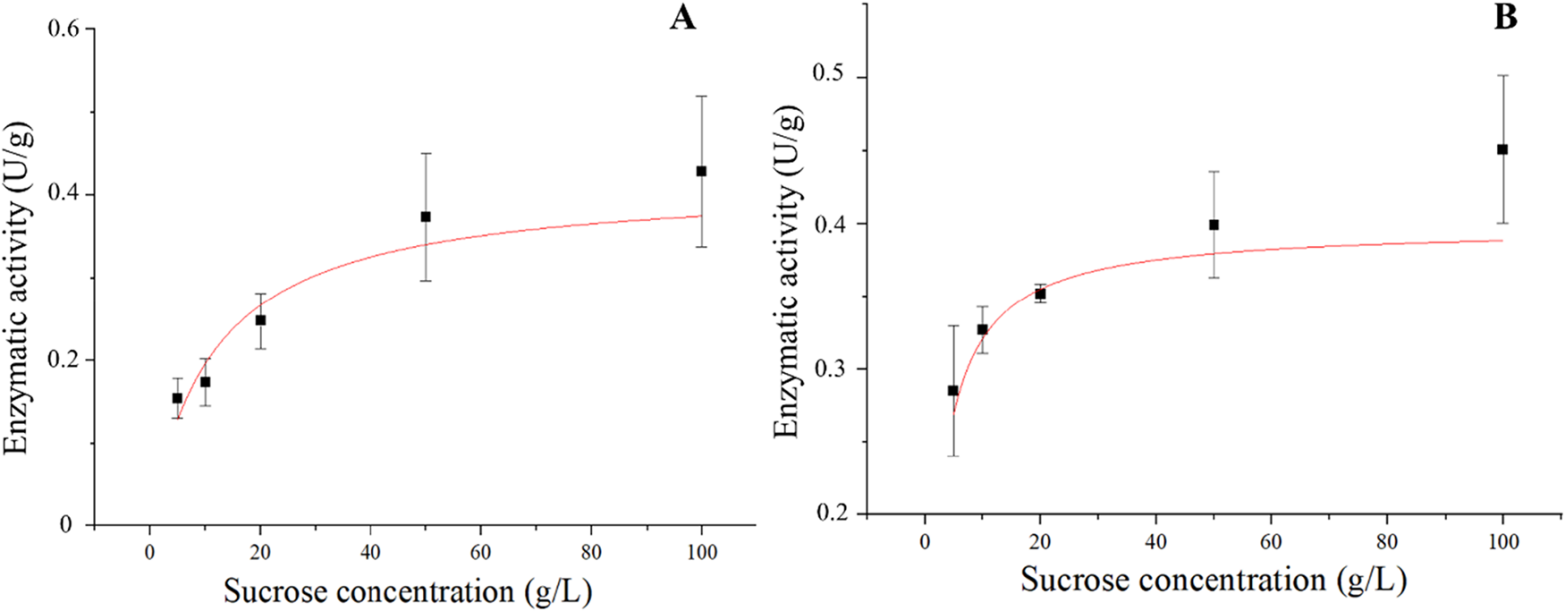
Michaelis–Menten model for the immobilization of the invertase from *B. tequelensis* (PP6) on SG (A) and PHB (B) with indication of the enzymatic activity (■), at 50 °C, with pH 5.0, for 8 h.

Valerio et al.^66^ noticed an increase in the *K*_m_ value after invertase immobilization on chitosan nanoparticles, demonstrating that immobilization reduced the affinity of the enzyme for the substrate, but it also retained the maximum capacity of invertase conversion. Szymańska et al.^67^ evaluated the kinetic parameters of sucrose hydrolysis after invertase immobilization on mesoporous cellular foam, observing 47.42 mM for *K*_m_ and 2.40 mM/min for *V*_max_. On the other hand, Hakkoymaz and Mazi^68^ found *V*_max_ of 13.04 μmol/min and *K*_m_ of 4.5 mM for invertase immobilization on poly­(*N*-vinylpyrrolidone-*co*-butyl acrylate-*co*-*N*-hydroxymethyl acrylamide) with sucrose concentration varying between 3 and 30 mM. These studies demonstrate that a higher affinity and efficiency for sucrose hydrolysis will vary according to the supports and methods used.

### Fructose Production

The enzyme immobilized on SG presented higher fructose production as a function of the reaction time (Figure 8), reaching a maximum concentration of 0.0682 M (68.2 mM) after 8 h of reaction. In contrast, the enzyme immobilized on PHB maintained fructose concentrations close to 0.013 M (13 mM) throughout an 8 h reaction period.

**Figure 8:**
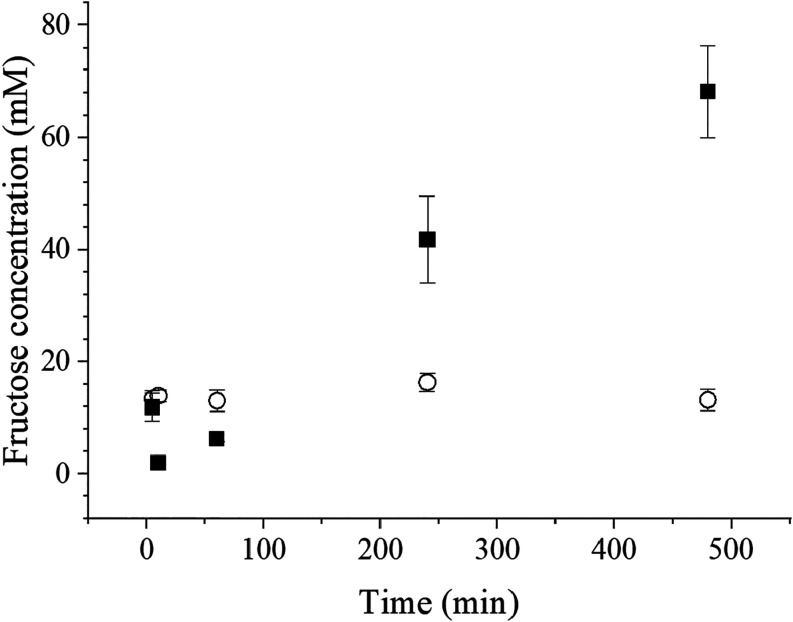
Fructose concentration as a function of time for enzyme immobilization on PHB (○) and SG (■) functionalized with glutaraldehyde, with substrate concentration of 100 g/L, for 8 h, at pH 5.0 and 50 °C.

The lower fructose production observed for PHB, despite its higher recovered activity (RA), may be associated with the hydrophobic character and semicrystalline structure of this polymeric support. Such properties may influence the enzyme orientation and substrate diffusion, potentially limiting active-site accessibility during prolonged hydrolysis. Conversely, the hydrophilic and porous nature of silica gel may favor substrate diffusion and a more effective catalytic turnover over time.

Although the invertase immobilized on PHB presented higher RA immediately after immobilization, cumulative fructose production over time was lower compared with SG. It is important to emphasize that RA reflects catalytic activity measured under controlled initial assay conditions, whereas fructose production during prolonged hydrolysis depends on additional factors such as substrate diffusion, enzyme orientation, and microenvironmental effects, which may limit the effective catalytic turnover in immobilized systems.^41,16,48^

Because of the hydrophobic and semicrystalline nature of PHB, enzyme immobilization may occur predominantly on external surfaces and may influence enzyme orientation or partially restrict substrate accessibility. In addition, reduced internal porosity and microenvironmental effects may contribute to diffusion limitations during the reaction. In contrast, the hydrophilic and porous structures of silica gel may facilitate substrate diffusion and improve effective catalytic turnover over extended reaction periods. Therefore, a higher RA does not necessarily imply a higher cumulative product formation, highlighting that initial catalytic activity and overall process performance are governed by distinct factors in immobilized enzyme systems.

It should be emphasized that these results represent the first report of fructose production using an immobilized invertase produced by a bacterial strain isolated from Amazonian fruit, contributing to the valorization of regional microbial biodiversity.

### Operational Stability of the Immobilized Enzyme

Figure 9 presents the enzymatic activity of the biocatalysts during their reuse in six consecutive reaction cycles. The invertase immobilized on SG presented the greatest activity retention in the last reaction cycle (approximately 20%). Nonetheless, this enzyme was capable of maintaining more than 50% of the initial activity until the third consecutive cycle. On the other hand, the invertase immobilized on PHB maintained 30% of its activity by the second cycle, which gradually decreased up to approximately 5% by the sixth cycle. These results indicate that the invertase immobilized on SG presents a significantly superior operational stability to the invertase immobilized on PHB, with large potential of reuse for up to 3 consecutive 1 h reaction cycles. This behavior can be attributed to the presence of stronger bonds in the invertase-SG complex induced by the functionalization with glutaraldehyde, which may also be associated with the lower RA presented by this biocatalyst, possibly due to conformational changes in the three-dimensional structure of the enzyme.^69^

**Figure 9:**
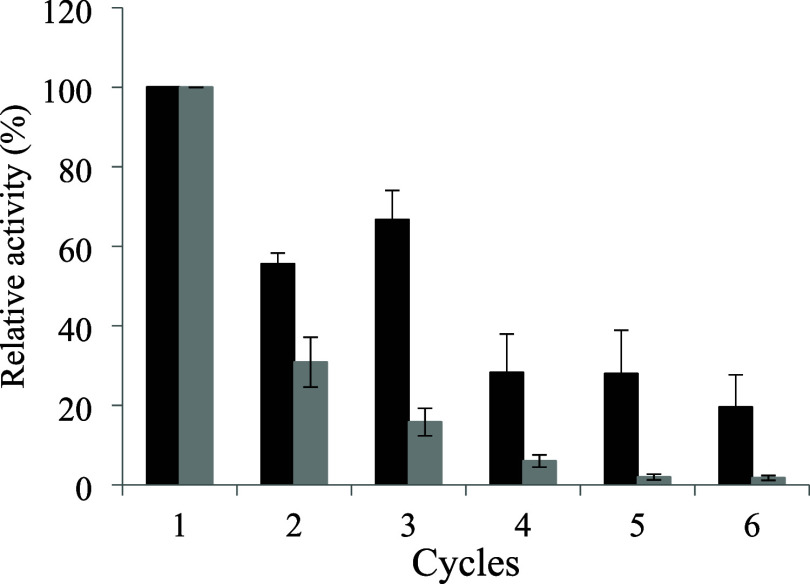
Operational stability of the invertase from *B. tequilensis* (PP6) immobilized on SG (■) and PHB (gray box) for 6 cycles.

After immobilization on PHB, the lipase studied by Binhayeeding et al.^40^ retained 50% of its initial activity for 15 cycles. Miranda et al.^70^ reported operational stability of approximately 70% of the initial activity after five reaction cycles, for the lipase from *Thermomyces lanuginosus* immobilized on mesoporous PHB particles. Mishra et al.^42^ evaluated the reuse of the enzyme immobilized on a silica-based support for 10 cycles. After 8 cycles, the immobilized enzyme presented 54% of activity, whereas the other support, also based on silica, presented 84% of activity, justifying the decrease in this percentage because of the desorption of the enzyme caused during washing. Rasbold et al.^7^ immobilized an invertase in calcium alginate, which presented above 50% of residual activity after 9 cycles, and 26% of residual activity after 12 cycles.

These findings demonstrate that the immobilized bacterial invertase, particularly the derivative obtained with SG, presents stability, reusability, and catalytic efficiency, making it a promising biocatalyst for sustainable invert sugar production.
